# Enhancing the design of voting advice applications with BERT language model

**DOI:** 10.3389/frai.2024.1343214

**Published:** 2024-08-06

**Authors:** Daniil Buryakov, Mate Kovacs, Uwe Serdült, Victor Kryssanov

**Affiliations:** ^1^e-Society Laboratory, College of Information Science and Engineering, Ritsumeikan University, Osaka, Japan; ^2^Digital Governance Systems Laboratory, College of Information Science and Engineering, Ritsumeikan University, Osaka, Japan; ^3^c2d, Center for Democracy Studies Aarau (ZDA), University of Zurich, Zurich, Switzerland

**Keywords:** voting advice applications, open data, topic modeling, language model, recommender system

## Abstract

The relevance and importance of voting advice applications (VAAs) are demonstrated by their popularity among potential voters. On average, around 30% of voters take into account the recommendations of these applications during elections. The comparison between potential voters' and parties' positions is made on the basis of VAA policy statements on which users are asked to express opinions. VAA designers devote substantial time and effort to analyzing domestic and international politics to formulate policy statements and select those to be included in the application. This procedure involves manually reading and evaluating a large volume of publicly available data, primarily party manifestos. A problematic part of the work is the limited time frame. This study proposes a system to assist VAA designers in formulating, revising, and selecting policy statements. Using pre-trained language models and machine learning methods to process politics-related textual data, the system produces a set of suggestions corresponding to relevant VAA statements. Experiments were conducted using party manifestos and YouTube comments from Japan, combined with VAA policy statements from six Japanese and two European VAAs. The technical approaches used in the system are based on the BERT language model, which is known for its capability to capture the context of words in the documents. Although the output of the system does not completely eliminate the need for manual human assessment, it provides valuable suggestions for updating VAA policy statements on an objective, i.e., bias-free, basis.

## 1 Introduction

Voting Advice Applications (VAAs) are online civic tech tools used by potential voters primarily during an election campaign. They provide users with recommendations on political parties that are closest to their own political leanings. A VAA can be seen as a recommender system where users are asked to express their agreement or disagreement with a set of political statements, for example, on a 3-point or 5-point Likert scale (see collaborative filtering (Aljunid and Dh, 2020)). The parties' responses in the VAA are established beforehand and stored in the system. With the user's input for each policy statement, a distance measure is calculated for each of the policy items. Thus, the user's political preferences are compared with those of political parties, using a matching algorithm (Mendez, 2017).

**Figure 1:**
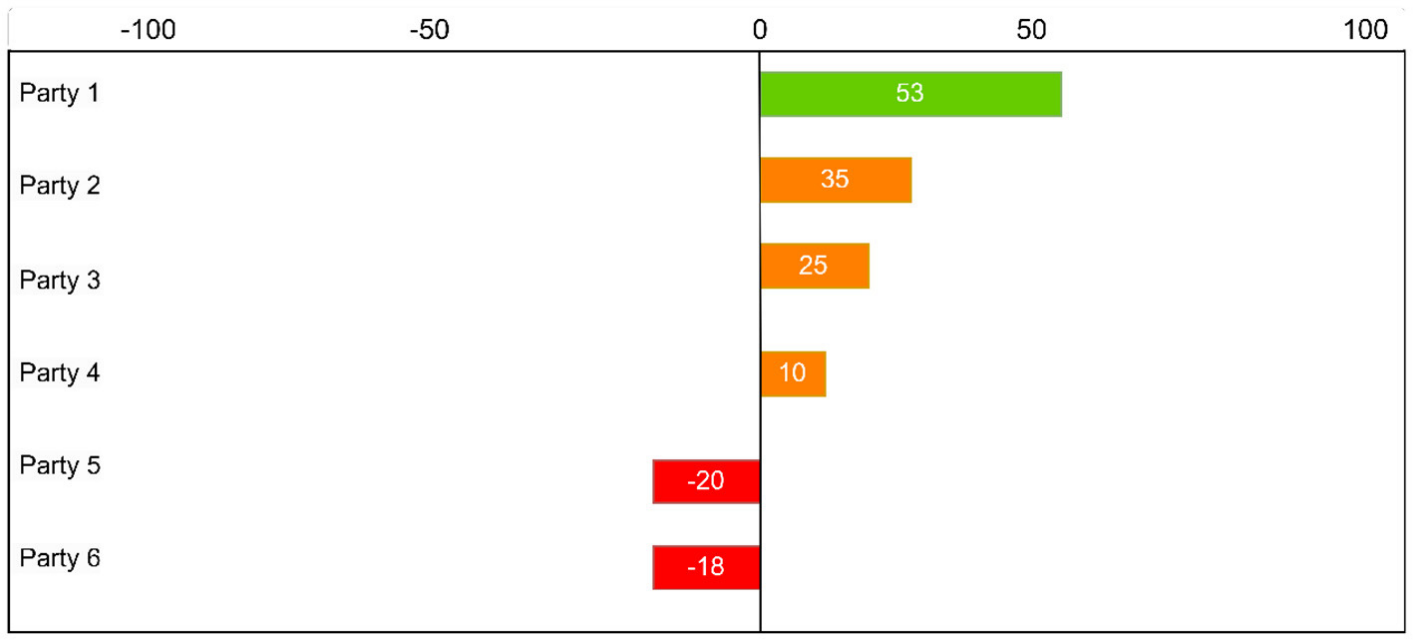
An example of the “overlap” in political views between a user and parties generated by a VAA, scaled on [–100, 100].

**Figure 2:**
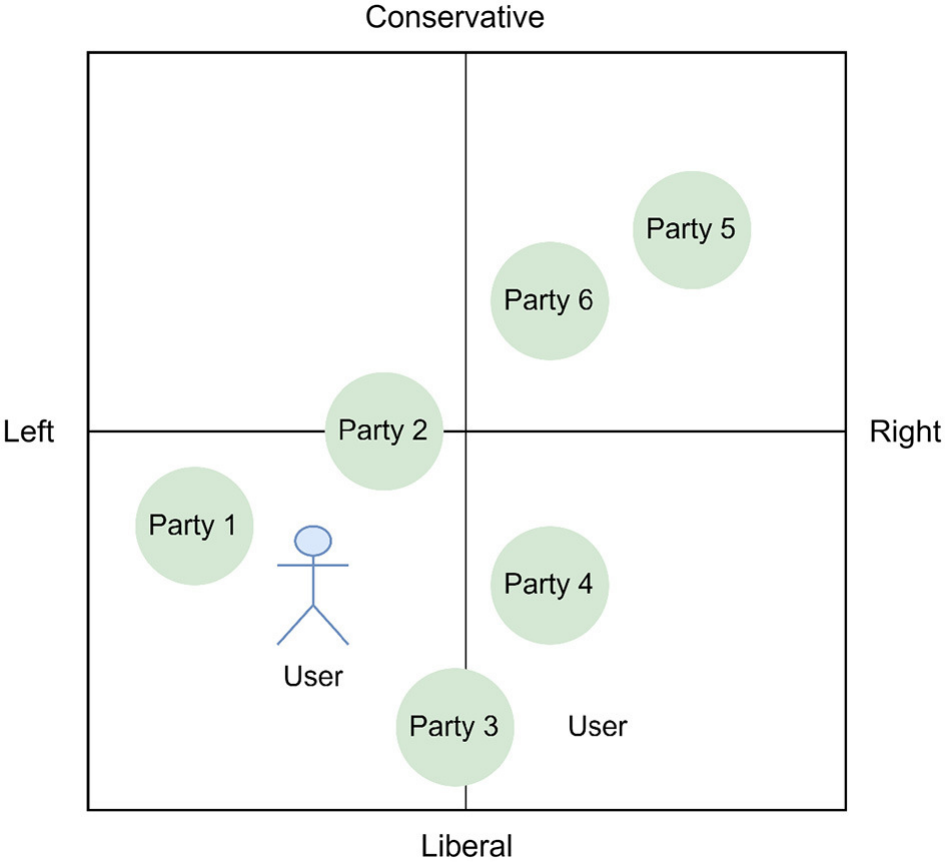
User and parties in a two-dimensional political space from a VAA with liberal-conservative social values and an economically left-right axis.

After the distance between the user's and each party's answers is calculated, the user can assess her or his “overlap” with each party in the corresponding two-dimensional ideological space (see Figures 1, 2). Therefore, VAAs provide the users with a time-efficient way to learn about their own political views and to form an understanding of the current electoral competition in any given constituency (Fossen and van den Brink, 2015; Holleman et al., 2016).

The deployment of VAAs has increased significantly over the past twenty years. Starting with a paper version in the Netherlands, VAAs soon emerged worldwide as online applications. For example, currently, between 10 and 40% of the electorate use a VAA in the weeks leading up to election day in various European nations (Garzia and Marschall, 2012; Germann and Gemenis, 2019). Such popularity among the electorate can partly be attributed to the existence of a multi-party system that makes electoral choice a more difficult task. In Japan, people typically rely on general internet resources and mass media for information about political parties. For instance, the “Policy Square” platform, launched in 2013, is designed to help Japanese electors make more informed decisions by providing information on the positions of political parties on various policy issues. Another example is the “Minna no Seiji” (Everyone's Politics) launched by the Tokyo-based NPO “Public Mind.” However, VAAs are currently gaining popularity in Japan, where the variety of VAAs already exceeds that in the EU. This can be attributed to the fact that in Japan, VAAs are not yet a well-established phenomenon and are subject to less regulations. Prior to each election in the country, there would be five to six VAAs made available. They are usually administered by large media corporations (e.g., https://vote.mainichi.jp/26san/; https://sangiin.go2senkyo.com/). The critical role that VAAs play in educating the electorate has attracted the attention of researchers in various fields, such as political science, computer science, sociology, etc. The latter is also due to the large volumes of data VAAs produce, sometimes even across several countries (e.g., Reiljan et al., 2020 and Wheatley and Mendez, 2021), that became the “fuel” for more research on VAAs (Dalton, 2016; Bruinsma, 2021; Huijsmans and Krouwel, 2021; Moreno et al., 2022).

Another reason for the increased popularity of VAAs among the electorate is their time-saving ability. Many potential voters routinely use VAAs to bypass the tedious gathering of information on party positions across relevant political issues and clarify their political vision in comparison with the party positions. VAAs can thus significantly influence the formation of people's political awareness, which is then reflected in their political orientation and attitudes, and ultimately has the potential to influence their political behavior (Tsutsumi et al., 2018; Munzert and Ramirez-Ruiz, 2021). Therefore, the choice of VAA policy statements, the matching algorithm, wording, and the way the results are presented are all essential components of the application and are the subject of constant research. However, the core part of VAAs that affects the final output is the policy statements (Lefevere and Walgrave, 2014; Isotalo, 2020). Naturally then, the policy statements must be carefully formulated and selected.

On the one hand, the policy statements must be relevant to the upcoming election, reflecting the current political situation in the country and the party spectrum. On the other, they must also capture the main political concerns of the electorate and possible critical issues that may arise during the electoral campaign (Garzia and Marschall, 2019). Formulating policy statements is, therefore, a critical, yet laborious and time-consuming process. It requires extensive knowledge of the electoral process and the political system in general. Statements are often chosen manually through iterative discussions between VAA designers and through an analysis of the relevant data. Generally, VAA designers learn the party views and main driving issues of the elections through questionnaires, surveys, party websites, and party manifestos. Annotating VAA statements for all political parties is an additional, tedious task that must be completed before a VAA is launched. To perform the annotation on behalf of political parties, annotators have to either personally contact the parties' representatives or possess the necessary expertise (Gemenis, 2015). However, since designers often lack time, they typically rely on party manifesto data that leads to a one-sided approach as it does not necessarily consider issues discussed by the citizens. In our view, additional data sources, such as e-petitions and social media data, should be utilized. The data potentially contains well-discussed topics and can serve as a gauge for determining the extent to which the views described in party manifestos genuinely reflect the interests of the citizens. Moreover, these data sources, along with party manifesto data, can be used for the verification of statements, ensuring the relevance and accuracy of past statements based on the current data. The inclusion of diverse data sources helps ensure that VAAs present a balanced view, reducing the risk of bias that might arise from relying on a single source of information. Utilizing additional data sources can also better address the cold start problem and enhance the quality of VAA statements. VAA statement quality is often evaluated *post hoc* by determining if it is possible to construct higher-order measurement scales. These scales involve creating complex tools that evaluate multiple aspects of the VAA statements. Such an approach can alleviate the cold start problem in VAAs and make it challenging to predict whether a statement will elicit the desired reaction from users (Germann et al., 2015; Germann and Mendez, 2016). VAA designers also have to be aware that specific issues included or excluded from a VAA can potentially benefit certain political parties. Moreover, all the factors mentioned above make the application susceptible to bias, which is often exacerbated by the time constraints involved.

The presented study aims to improve the effectiveness of VAA formulation and statement selection process using information technology. While the system proposed in the study would not eliminate the need for human analysis, the results of the experiments show that it would help VAA designers to save time and effort, and potentially reduce the bias in the process. A case study has been conducted using VAA statements, party manifestos, and social media data from Japan to investigate the effectiveness of the proposed system and to show how the approaches can be modified according to the characteristics of documents and the preferences of the VAA designers. Earlier results of the current work have been previously reported in Buryakov et al. (2022a) and Buryakov (2023).

The main contributions of the presented study are as follows:

- Development of an original system: a novel system for generating VAA design suggestions was developed and tested through a case study in a real-world scenario. The proposed system not only demonstrated an efficiency gain but also contributed to the development of a more robust empirical basis for a crucial aspect of a VAA—the selection and formulation of VAA statements.
- Utilization of diverse information sources: due to the efficiency gains achieved with the system, it became feasible to utilize both conventional sources of information, such as party manifestos, and unconventional sources, such as social media data. This inclusive approach allows for a more balanced reflection of both political entities' positions and voters' concerns within the VAA, moving away from a manifestos-only approach.
- Utilization of foreign VAA statements: the utilization of VAA statements from other countries helps designers capture overlooked or emerging topics. This expands the VAA's scope, ensuring a more diverse and comprehensive reflection of political issues.
- Implementation of statement verification feature: the system's capability for statement verification against previous elections results using current politics-related data introduces a new approach to VAA design. This feature enables VAA designers to validate and update statements to ensure they remain relevant and reflect the latest political dynamics.

The rest of this paper is organized as follows: The next section introduces the concept of a VAA and related work on machine learning and natural language processing methods used in the study. Section 3 describes the proposed system in detail. Sections 4, 5 describe the data used in the case study and present experiments made. Section 6 reveals the obtained results. Finally, in Sections 7, 8, the main findings and a discussion, limitations, and future work are provided.

## 2 Related work

Natural language processing and machine learning are examples of computational tools that have been successfully applied in civic tech and policy domains in recent years. For example, in Hagen (2018), using Latent Dirichlet Allocation (LDA) topic modeling, petition data was processed and evaluated to classify citizens' proposals. The developed framework enabled the generation of human-interpretable topics that closely matched the results of independent content analysis. Thus, the framework could aid in enhancing democratic decision-making processes and analyzing public opinion regarding political issues. Romberg and Escher (2022) developed a supervised machine learning method that could correctly identify topic categories of citizens' proposals. As a result, this approach reduced the time needed for manual labeling. In a different study (Di Cocco and Monechi, 2022), a method based on supervised machine learning for measuring levels of parties' populism in six European countries was developed. The authors analyzed party manifestos over nearly two decades using a random forest classification algorithm and identified the level of populism without resource-intensive human-coding processes.

In recent years, topic modeling, which is an unsupervised machine learning method, has been widely used for discovering topics in a set of documents. In Silva et al. (2021), 1,313 comments on two bills were analyzed to identify topics in Brazilian comments on legislation bills. The authors illustrated the topic modeling applicability to uncover latent topics to improve civic engagement. In Anwar et al. (2021), topic modeling was used to examine 12 million tweets about the US election posted on the “QAnon” platform between August and September, 2020. A list of predominant topics with the extensive support of one candidate was thus found. Topic classification methods were also used by Arana-Catania et al. (2021) to address the problem of information overload. The authors were able to categorize citizens based on their individual preferences after analyzing comments posted in response to proposals made on the CONSUL platform.

Bidirectional Encoder Representations from Transformers (BERT), developed by Google researchers in 2018, is a language model recently often used for text similarity assessment, text summarization, and fake news detection. In Gaglani et al. (2020) the authors conducted an analysis of the reliability of claims in instant messaging apps. BERT helped to determine the degree of similarity between claimed messages regarding significant events and articles from trusted news sources on the same topic. Giachanou et al. (2020) devised a technique for detecting fake news that incorporates visual, linguistic, and semantic inputs. Images from various articles were vectorized, while textual information was retrieved from article headlines using a BERT model. As a result, the built system could make predictions of the legitimacy of the online content. The proposed approach outperformed the baseline model. BERT was also utilized by Shirafuji et al. (2020) for the summarization of political utterances. The authors trained the developed summarizer on 27,078 statements from the Tokyo Metropolitan Assembly dataset. It was concluded that although the results obtained in the study were mostly unsatisfactory, the system demonstrated the potential usefulness, as its performance could be improved with additional training on a news corpus.

Despite the fact that machine learning and natural language processing made it possible to categorize enormous quantities of data, allowing information to be updated and summarized in a more efficient way, there has been little research on opinion mining for VAA design. Few recently published studies include Terán et al. (2017) and Terán and Mancera (2019), where the authors tried to implement dynamic candidate-party profiles into VAA to minimize bias in the recommendation process to potential voters. Through the sentiment analysis of Twitter data, the authors found the most discussed political topics and their importance to election candidates and parties that potentially can be incorporated into a VAA.

## 3 Methods

### 3.1 Framework

**Figure 3:**
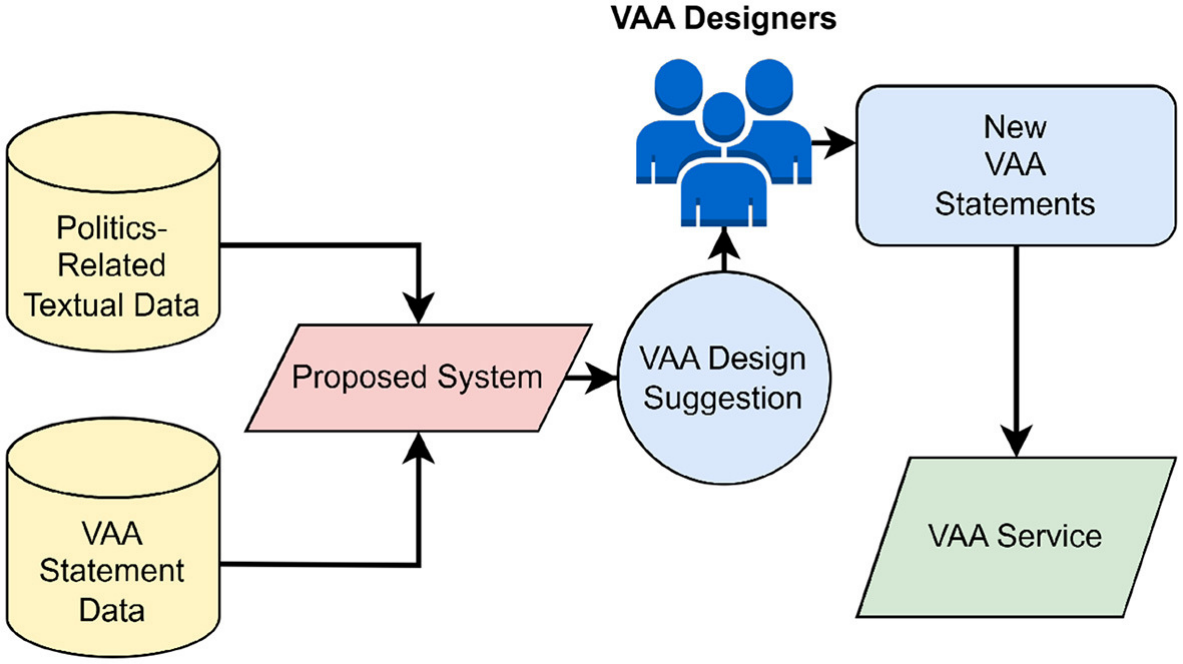
Framework for the proposed system.

The proposed system represents a natural evolution of the research aimed at enhancing VAA development and quality through the use of information technology. It produces suggestions based on the VAA statements from the previous elections and any current politics-related textual data. The choice of the inputs is ultimately arbitrary and can only be assessed *post hoc*, e.g. as in Gemenis (2015) and Germann et al. (2015). The system's overall framework is depicted in Figure 3. Before launching the VAA service on a web-based platform where it would be accessible to users, the designers may consider suggestions generated by the system for formulating the final version of the statements. Note that text-based suggestions are the supplemental tool for the designers. Hence, again, the suggested solution does not strive to automate the work of VAA designers fully but to support them by reducing the amount of time needed to formulate VAA statements for the upcoming elections.

### 3.2 Proposed system

The general approach of the system begins by organizing politics-related textual data into distinct semantic groups using topic modeling. Subsequently, a cosine similarity measure is applied, with a threshold of 0.5, to focus on specific statement-topic groups. This selective process ensures that suggestions are provided only for statements that are relevant to current data, thereby avoiding suggestions for all statements indiscriminately. Finally, within the selected groups, suggestions are made based on the highest cosine similarity score between documents and the selected statements. The methods employed in the system are adapted based on the length and number of documents, as well as on the preferences of VAA designers. Although two approaches are discussed in the paper, only approach 1 was utilized in the current paper.

**Figure 4:**
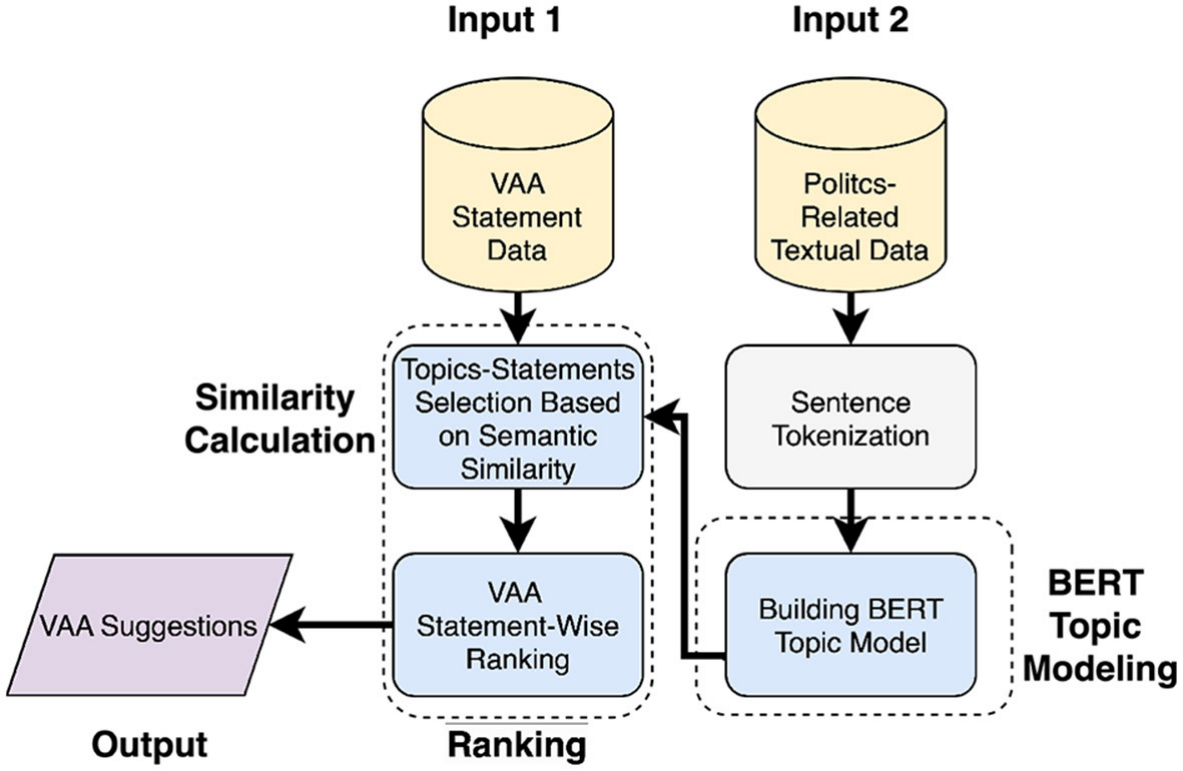
Processing flow of the proposed system (approach 1).

Figure 4 (approach 1) shows the system's processing flow, where all politics-related documents are processed for the similarity calculation step. In the beginning, BERT topic modeling is applied to produce topics, where each of the topics includes semantically connected sentences stemming from the textual data. When there are a few documents encompassing multiple topics, it is advisable to split these documents into individual sentences prior to the topic modeling stage. This is because BERT-based topic modeling typically associates each document with a single topic, and effective topic modeling cannot be conducted with only a limited number of documents. Splitting documents into sentences will produce a list of documents each containing one sentence. After topic modeling, the semantic similarity between all topics and every VAA statement is calculated by averaging the cosine similarity scores of sentence vectors from particular topics and a VAA statement vector. Vectors for cosine similarity computation are generated using the sentence-BERT model, which is known to outperform the original BERT model in terms of computational efficiency (Reimers and Gurevych, 2019). If the semantic similarity score between the documents related to the topic and statement exceeds 0.5, these are selected for the final output. With this method, suggestions are produced only for statements related to the documents. A score of 0.5 is used as a threshold, since a score above it would demonstrate a statement-topic relevance. In the end, sentences with the highest individual similarity scores are extracted from the pool of selected topics and used as suggestions for the selected VAA statements.

**Figure 5:**
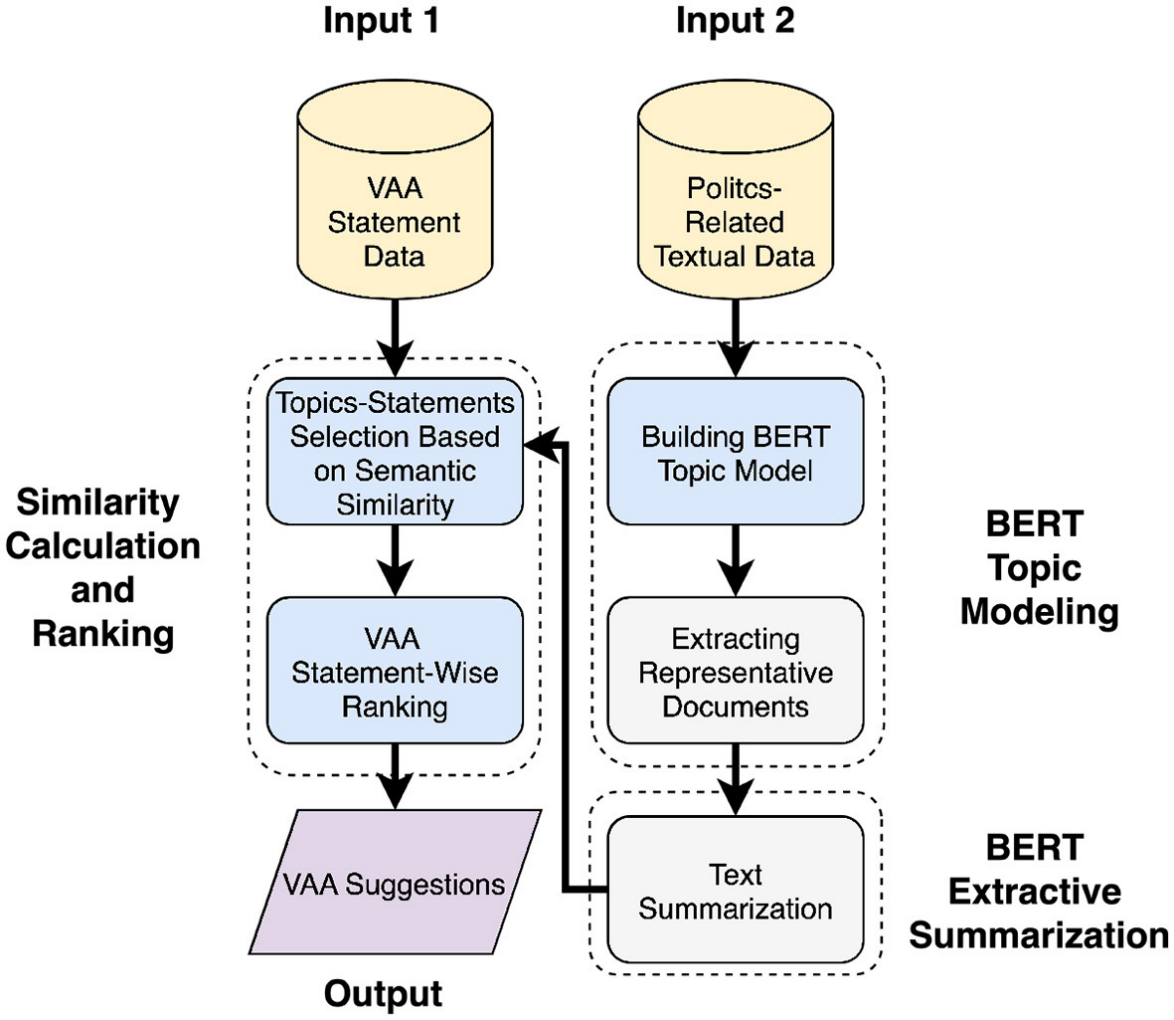
Processing flow of the proposed system (approach 2).

Figure 5 (approach 2) shows the system's processing flow adjusted for ranking suggestions originated from the summaries of the most representative documents. Text summarization and extraction of the most representative documents are optional methods of the system since this approach reduces the amount of data while keeping only the most representative documents and reducing the memory usage in the similarity calculation step. Applying text summarization is appears reasonable when there are many long documents that would be troublesome to read in the output. After the topic modeling is conducted, the most representative documents are extracted for summarization. In the end, the semantic similarity between summaries and VAA statements is calculated to produce a ranked list of statement-wise suggestions. These are then made ready for the further use by domain experts and VAA designers. A detailed description of the methods applied is outlined below.

### 3.3 BERT topic modeling

The pre-trained BERT language model constitutes one of the foundational elements of the study's methodology and is first applied for topic modeling. Topic modeling is an unsupervised machine learning method for extracting topics from a group of documents. Popular topic modeling techniques, such as LDA (Blei et al., 2003), use probabilistic models, and process documents as “Bag-of-Words.” LDA, however, has a sparsity issue (Lu et al., 2021) and ignores word context and ordering (Silveira et al., 2021).

A significant strength of the BERT language model (Devlin et al., 2019) is its capability to generate contextualized word embeddings. A word embedding is a numerical vector that represents a word in a continuous vector space, where semantic similarity is gauged by measuring the distance between word vectors. By averaging the values of token embeddings, this context-aware language model can produce word embeddings and sentence embeddings. Tokens, whether they take the shape of words, punctuation, or numbers, represent a single piece of information in natural language processing. In contrast to static models like Word2Vec (Mikolov et al., 2013), BERT can process inputs in both directions and produces vectors, depending on their actual context. The BERT model applied in the study contains 12 layers of attention heads and has an output vector size of 768 dimensions. The attention heads allow the model to focus on different parts of the input data that improves its ability to understand the context and relationships between words in a sentence.

BERT topic modeling (Grootendorst, 2022) performs as follows: 768-dimensional vectors derived for each document are reduced using Uniform Manifold Approximation and Projection for Dimension Reduction (UMAP). In the next step, the Hierarchical Density-Based Spatial Clustering of Approximation with Noise Algorithm (HDBSCAN) (McInnes and Healy, 2017) is applied to cluster semantically related embeddings. The advantage of the algorithm is that it performs well even when the clusters have complex shapes and a significant amount of noise. HDBSCAN also can reduce the amount of data. It is achieved by retrieving the most representative documents from each cluster (topic), known as HDBSCAN exemplar points or core points. Including this optional method in the proposed system would significantly reduce the computational load, keeping only the most important documents.

Class-Based Term Frequency-Inverse Document Frequency (C-TF-IDF) scores are then calculated for all words to assess their importance within clusters (topics). C-TF-IDF of term *x* within class *c* is determined as: ${C-TF-IDF}_{x,c}={tf}_{x,c}\times \log\left(1+\frac{A}{f_{x}}\right)$, where *tf*_*x, c*_ is the frequency of word *x* in class *c*, *f*_*x*_ is the frequency of word *x* across all classess, and *A* is the average number of words per class. The system can find the most relevant words for each topic with C-TF-IDF. The higher the C-TF-IDF score, the more a word represents a particular topic. Although topic labeling is not required, retrieving the most relevant words for each topic allows the system to evaluate topic models using a *Cv* score and to select the one with the most appropriate number of topics.

### 3.4 Topic model evaluation

The most challenging part in building a topic model is to choose the optimal number of topics. An optimal number of topics depends on the number of documents processed by the system, its contents, and other characteristics. The quantity of topics chosen affects the quality of a topic model and the consistency of topics. A conventional method for evaluating topic models is to manually evaluate their output. However, this is a costly and subjective approach (Shi et al., 2019).

To avoid relying only on subjective evaluation and to save the time of VAA designers, the proposed system provides the ability to build several topic models and to select the most appropriate model based on a quantitative metric. However, the objective quality assessment of the topic model quality is not mandatory within the system and may also be conducted manually if time constraints do not limit the VAA designers. Although there are many metrics for evaluating topic models, it was decided to employ the coherence measure (*Cv*), as according to Röder et al. (2015) and Syed and Spruit (2017), it is the most highly correlated metric with human judgments. *Cv* score is based on a sliding window, cosine similarity, Normalized Pointwise Mutual Information (NPMI), and a one-set segmentation of the most relevant, descriptive words which, in our study, were extracted using C-TF-IDF. In the *Cv* calculation step, each topic word is compared to the entire collection of topics. A sliding window is used to determine whether two terms co-occur. For each topic's *N* most probable words, a word vector of size *N* is constructed, with each cell containing the NPMI between that word and other words in the topic. Then, each topic's word vectors are aggregated into a single topic vector. The *Cv* score is calculated using the average cosine similarity between each topic word and its topic vector.

### 3.5 BERT extractive summarization

**Figure 6:**
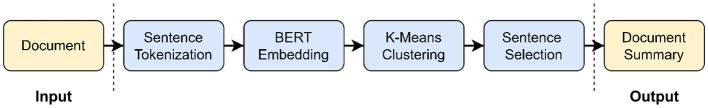
Processing flow of the BERT extractive summarizer.

The BERT extractive summarizer (Miller, 2019) can be used to summarize the most representative documents retrieved with the HDBSCAN method, thus reducing the workload of designers in formulating the statements. Extractive summarization, in contrast to abstractive summarization methods, does not create any new text but produces a summary with the most important sentences of the original document. Figure 6 depicts the summarization algorithm. Sentences from the document are embedded using BERT language model, and a *k*-means algorithm is used to identify sentences closest to the centroids. The length, i.e., the number of sentences, is determined manually based on the size of the original input text.

### 3.6 Similarity calculation and ranking

Semantic similarities between selected VAA statements and documents (approach 1) or between all VAA statements and summaries of the most representative documents (approach 2) are measured by calculating the cosine similarity between the statement-document vectors on the interval of [0,1]. Since the original BERT model was not trained for similarity tasks and tends to perform poorly in this aspect (Li et al., 2020), the sentence-BERT model was deployed, which generates comparable fixed-sized sentence vectors that are essential for the comparison. The closer the cosine of the angle approaches 1, the greater the similarity between the texts. This metric is initially used to identify topic-statement pairs that exceed a predefined cosine similarity threshold. Subsequently, it is used to rank documents, prioritizing those with the highest similarity scores for the VAA statements that have surpassed the threshold. Approach 2 was applied in a different case study (Buryakov et al., 2022b), further illustrating that the system is not limited to specific technologies.

## 4 Data

A case study was conducted using VAA data, party manifestos, and YouTube comments to produce suggestions for VAA designers from Japan. The following considerations dictated the choice of the datasets. Party manifestos, being the conventional source from the political parties, tend to have a formal tone that often omits radical views. On the contrary, social media platforms like YouTube offer a space where anyone can express their opinions quite freely, providing a rich and diverse range of perspectives that are invaluable to VAA designers. The proposed system expands the variety of datasets that can be used for VAA design, moving beyond the conventional reliance on a manifestos-only approach. It also enables the analysis of how well the past VAA statements align with the current data, thereby facilitating their verification. Suggestions were produced separately for each dataset. It is worth noting that the proposed method is applicable in other countries where VAAs are used, and party manifestos with social media data or something similar, such as online petitions, are available.

### 4.1 VAA data

The policy statements from two European and six Japanese VAAs were manually extracted and used as VAA input data for the system. VAA statements from the European VAAs were added to expand the policy space. Opening the policy space with statements from outside of Japan enabled the discovery of potentially important concerns that were not initially expressed in the Japanese VAAs. This might help VAA designers raise new ideas about what issues are currently important and should be covered in VAA policy statements. European and Japanese VAA statements were created for the 2019 European Parliament elections and 2021 Japanese Upper House elections, respectively, from the following VAAs: EUandi (Reiljan et al., 2020), EUvox (Gemenis et al., 2019), Zero Senkyo, Shimotsuke Shimbun, Japan Choice, FokusJapan, Asahi Shimbun and Mainichi Shimbun. In total, there were 135 and 50 statements covering the most important political issues in Japan and the EU, respectively. An example of statements from each VAA is presented in Table 1.

**Table 1:** A translated example of the utilized VAA policy statements.

**Voting advice application**	**Policy statement**
EUandi	Immigrants from outside Europe should be required to accept our culture and values.
EUvox	The number of public sector employees should be reduced.
Zero Senkyo	Japan should stop the selective surname system.
Shimotsuke Shinbun	Do you think the consumption tax rate should be temporarily reduced to 5%?
Japan Choice	Do you think nuclear power plants should continue to operate?
FokusJapan	Pension payments should be gradually reduced.
Asahi Shimbun	Japan's defense capabilities should be strengthened.
Mainichi Shimbun	Do you agree or disagree with a female member of the royal family becoming emperor?

While most VAAs elicit an agreement or disagreement response from the users to policy statements, some make questions with options for categorical or agreement-based responses. Note that the language used to formulate policy statements utilized in VAAs should be clear and straightforward enough to be understood by all groups of people. To avoid confusing the users, VAA designers should not employ double negations, acronyms, foreign words, or complicated numbers. Presently, most users examine a VAA utilizing smartphones, making the need for concise wording even more critical. This fact emphasizes how difficult the task of developing VAA statements is in practice.

### 4.2 Party manifestos

Party manifestos uploaded two weeks before the 2021 Japanese Upper House and the 2022 House of Councilors elections (Hino et al., 2022) were manually retrieved from the official party websites by a research group on Japanese elections from Waseda University. Manifestos are essential to VAA designers for statement formulation and for potential voters to understand the differences between parties. Party manifestos originated from the following political parties: *Japan Innovation Party (known as Ishin), Liberal Democratic Party, Reiwa Shinsengumi, Democratic Party for the People (DPP), Japanese Communist Party (JCP), Komeito, Constitutional Democratic Party of Japan, Social Democratic Party of Japan and NHK Party*.

### 4.3 YouTube comments

YouTube comments from the 18 most popular Japanese channels related to politics and economics were employed as social media data for the case study (see Table 2). The channels' total subscriber number adds up to ~7,629,000. A total of 6,251,246 comments from 27,270 videos were retrieved using YouTube API.

**Table 2:** The YouTube channels used in the case study.

**Channel name**	**Subscribers**
橋洋一チャンネル	706,000
KAZUYA Channel	675,000
立花孝志のターシーch【NHKの裏側】	537,000
SakuraSoTV	534,000
【公式】竹田恒泰チャンネル 2	511,000
及川幸久THE WISDOM CHANNEL	492,000
文化人放送局	488,000
上念司チャンネルニュースの虎側	475,000
武田邦彦テレビじゃ言えないホントの話!	420,000
デイリーWiLL	411,000
フィフィ（FIFI）	385,000
ChGrandStrategy	312,000
青山繁晴チャンネル•ぼくらの国会	303,000
藤井厳喜の『ワールド・フォーキャスト』	287,000
大紀元エポックタイムズ・ジャパン	286,000
古是三春篠原常一郎	283,000
令和タケちゃん【撃退・報道系YouTuber】	263,000
松田政策研究所チャンネル	261,000

However, in order to use data relevant to the upcoming elections, and given that issues of public concern may change over time, only comments from videos uploaded between January 2021 and July 2022 (17,773 in total) were kept in the YouTube comment dataset (3,998,590 comments). Out of all the comments, many had just one sentence and, for that reason, were of no use. Further refining the dataset, comments with fewer than 40 characters were excluded based on Kanji Hatano's 1960s proposal, who suggested that the optimal length for a sentence written in Japanese should be between 40 and 60 characters (Hatano, 1965)—a view supported by contemporary academic consensus (Katagiri, 2022). After removing duplicates and overly short comments, the dataset was reduced to 2,126,837 comments for the case study.

With more than two billion active users and millions of individuals expressing their opinions, YouTube comments can be a valuable source of information for VAA designers. Compared to other types of data, YouTube comments have their uniqueness. For example, unlike party manifestos which are texts of official announcements and messages viewed by the public, YouTube comments can be considered as uncensored political ideas from the electorate side. This could also serve as a source of information about citizens' opinions, which VAA designers might utilize both for formulating new statements and verifying the existing ones. An appealing component of the given social media is that users there feel free to publish comments without their identity being detected. On the other hand, popular sources of public opinions, such as e-petitions (e.g. see Buryakov et al., 2022b), are more controlled, as the public administration usually formally examines the ideas in a “verification phase” before opening them for voting. Therefore, YouTube comment data differs from other datasets due to the fact that users who upload them feel less controlled and safer to express unpopular opinions.

## 5 Experiments

**Figure 7:**
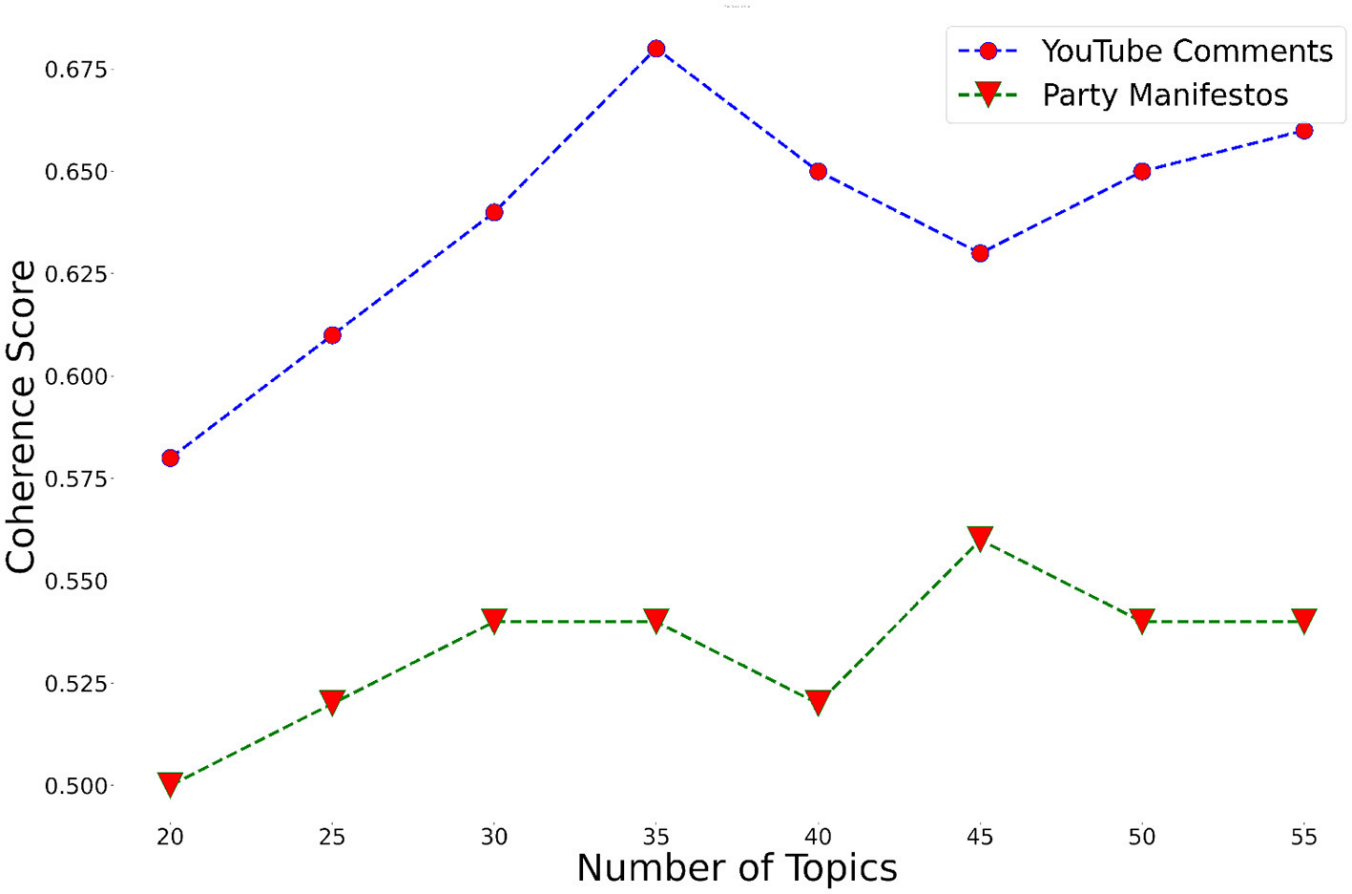
Topic coherence score of topic models built based on the party manifesto and YouTube comment datasets.

Prior to BERT topic modeling, party manifestos from the nine Japanese political parties were split into sentences. The whole manifesto corpus contained 13,408 sentences. After removing identical sentences and sentences with less than 40 characters, 9454 sentences remained. The average length of one sentence was 71.8 characters. Eight topic models were built to choose the most optimal one, changing the number of topics *k* from 20 to 55, with an increment of 5. The number of descriptive words, i.e. words that represent a particular topic, was set to 10. The model with 45 topics was finally selected based on the coherence score (see Figure 7).

Instead of assessing topics manually, the quality of topic models was measured by using the *Cv* coherence score metric. The higher the coherence score and thus the quality of the topic model, the better descriptive words represent topics. Since topic labels are not used by the VAA designers, topic labeling was not conducted. The applied topic modeling technique assigned each document to a particular topic. If a document could not be assigned to any topic, the document was considered an outlier. A total of 1,670 documents or almost 18% of the whole corpus were classified as outliers. As a result, the amount of data for the next processing was significantly reduced by keeping only the topically relevant parts of the corpus.

In order to select topics from which the most relevant sentences are extracted for the VAA statements, the semantic similarity between each topic and VAA statements was calculated. This was done by calculating the average cosine similarity between all documents of a topic and each VAA statement. Topics and VAA statements with cosine similarity scores above the threshold of 0.5 were selected. Since there were 185 statements from Japanese and European VAAs, and they often addressed similar issues and sometimes were exactly the same, only one of each identical or similar statement was manually retained in the final output. This approach ensured that the final set of statements was concise and free of redundancy, accurately representing the diverse topics addressed. In total, there were 26 statements to which topics were assigned. After this, the semantic similarity between sentences from the selected topics and VAA statements was calculated. Sentences were ranked according to the similarity scores. The system was set to output the top five suggestions for VAA designers as options to work with. A total of 130 suggestions for 26 original statements were produced. Five suggestions were considered to be what VAA designers can quickly go through. However, the number is arbitrary and can be set based on the available time and resources. Party names identifying the manifesto source of the sentences are also outlined in the final results so that VAA designers can comprehend the level of inter-party division toward the statements.

The methods used for party manifestos were also applied to YouTube comments. However, to avoid losing the context of comments that were mostly short, the sentence tokenization step was skipped, so that one comment was considered as one document. The sentence tokenization step, however, is necessary if there is a small number of documents with a large amount of text, as it was in the case of the party manifesto data. The average number of characters per comment was 114. Out of eight topic models, a model with 35 topics performed best in terms of coherence score (see Figure 7). A total of 425,365 comments (almost 20%) were classified as outliers since they were not assigned to any topic. The slightly higher number of outlier documents compared to the party manifesto data may be due to the prevalence of meaningless comments on YouTube. YouTube is generally infamous for hate speech, unconstructive criticism, and trolling, especially in politics-related videos. Further processing steps were the same as in the case of party manifestos; overall, the system produced 70 suggestions for 14 statements. Interestingly, the number of topics selected and statements produced show that party manifestos cover more topics compared to YouTube comments. One reason for this may be that in close elections, political parties need to address a variety of issues to attract people from diverse social groups. In total, 24 unique statements were derived from party manifestos and 12 from YouTube comments, with only two overlapping statements between the datasets. This indicates a significant disparity between the issues users discussed on social media and those political parties prioritized in their manifestos. However, this discrepancy could also be attributed to the difficulty in assigning comments to topics due to the limited information of the comments.

## 6 Results and discussion

Using approach 1, the proposed system separately produced the top five suggestions for the selected VAA policy statement from both the party manifesto and YouTube comment datasets. Table 3 shows suggestions for a particular VAA statement extracted from the party manifesto data. The similarity score has been omitted here as it is not important for understanding the specific result being discussed.

**Table 3:** A translated example of system suggestions for a VAA statement produced based on party manifesto data.

**VAA statement**
Higher education should be completely free of charge.
**System suggestion 1**
*[JCP]* We are aiming for free education, halving tuition, abolishing entrance fees, and eliminating school lunch fees—halving tuition at universities and vocational schools, and making them free in the future.
**System suggestion 2**
*[Japan Innovation Party]* Zero educational burdens for the next generation of children, all education such as early childhood education, high school, university, etc., so that we can receive an equal quality education regardless of the financial situation of the family.
**System suggestion 3**
*[Reiwa Shinsengumi]* We will create a society where people can go to graduate school for free without owing debt if students are willing to learn.
**System suggestion 4**
*[Komeito]* Expand the scholarship program and tuition reduction/exemption to middle-income families, including families with multiple children and students studying science, engineering, and agriculture, who especially need to reduce their burden, so that anyone can enter university if they wish, regardless of their family's financial situation.
**System suggestion 5**
*[DPP]* Reduce tuition fees for higher education, including universities and graduate schools, and extend non-repayment scholarships to average families.

The statement takes up the current political debate in Japan concerning the abolition of tuition fees in higher education. This issue has received much attention in recent years and is a part of the ongoing debate about the cost of education, which has been characterized as “expensive” compared to other industrialized nations. Some political parties, including the Japan Innovation Party, the Democratic Party for the People, and the Reiwa Shinsengumi, called for free or nearly free higher education. Several opposition parties advocated for a tuition-free system, while the Liberal Democratic Party (LDP) and Komeito advocated reducing private school tuition and increasing the number of interest-free scholarships. As an evidence of the proposed system's ability to provide suggestions related to the existing political reality, the system developed concepts consistently with the aforementioned political context and, as a result, excluded the LDP from the final suggestions for the statement. The output highlights the usefulness of the proposed approach for VAA designers, demonstrating its capability to produce results that reflect a country's political discourse.

The produced results were forwarded to the FokusJapan group of VAA designers to aid in the statement formulation process for the July 10, 2022 Upper House election. This was done to evaluate the system in a real-world scenario. In the end, VAA designers came up with twenty policy statements, nine of which were made based on the suggestions produced by the system (see Table 4). The VAA designers made their choices completely independently, without any coordination with the authors of this article. Suggestions generated from party manifesto data primarily address issues related to economics. Some of the statements generated were identical to those used in the previous elections, suggesting that the proposed approach can not only recommend the implementation of new statements but also verify previously employed statements for reuse based on current data. One statement that passed the similarity threshold originated from the European VAA (Statement 7, in Table 4), indicating that this issue is intensely discussed in both Europe and Japan. The remaining eleven statements were added to the VAA based on the manual assessment of party manifestos.

**Table 4:** System's output statements used in FokusJapan.

**(Japanese VAA) Statement 1**
Economic activity should be prioritized over infection control of coronavirus.
**(Japanese VAA) Statement 2**
A carbon tax should be introduced to reduce greenhouse gases.
**(Japanese VAA) Statement 3**
Nuclear power plants should be decommissioned in advance.
**(Japanese VAA) Statement 4**
The consumption tax should be reduced to 5% or less.
**(Japanese VAA) Statement 5**
Taxation on the rich should be strengthened.
**(Japanese VAA) Statement 6**
The minimum wage should be raised to 1500 yen or more.
**(European/Japanese VAAs) Statement 7**
The amount of pension payment should be gradually reduced.
**(Japanese VAA) Statement 8**
Higher education should be completely free.
**(Japanese VAA) Statement 9**
The eligibility age to be elected should be lowered.

The system's output results show the potential to cut down on the amount of time and effort that VAA designers have to put into their work. The results, which include the stances of political parties on various issues, facilitate the removal of certain statements. Specifically, if there is consensus among all parties regarding a particular issue, meaning the statement does not differentiate between the parties, then it may be removed since it is useless for a VAA. Furthermore, the proposed approach allows for the measurement of a problem's or topic's significance to a particular party based on the similarity score and its ranking. Manually assessing the importance of such critical issues would be both time-consuming and laborious. The proposed aproach proves especially valuable in Japan, where VAA designers typically have only few weeks during the election campaign period to develop statements. Compared to previous results, the statements derived from the YouTube comments focus more on politics rather than economics (see Table 5).

**Table 5:** VAA statements produced by the system based on YouTube comments.

**(European VAA) Statement 1**
Mainstream Islam is compatible with British values.
**(Japanese VAA) Statement 2**
The laws should be changed so the government can restrict private rights and impose lockdowns.
**(Japanese VAA) Statement 3**
Pressure should be prioritized over dialogue with North Korea.
**(Japanese VAA) Statement 4**
Do you agree that Japan should have the “enemy base attack capability” that can strike the opponent's base before being attacked?
**(Japanese VAA) Statement 5**
Do you think China is a threat to Japan or is it a partner?
**(Japanese VAA) Statement 6**
Do you agree that Japan's defense spending should be increased?
**Japanese VAAs) Statement 7**
Japan's current foreign policy toward South Korea should be stronger.
**(Japanese VAA) Statement 8**
Japan should join the Treaty on the Prohibition of Nuclear Weapons.
**(Japanese VAA) Statement 9**
Should the US-Japan Alliance be maintained?
**(Japanese VAA) Statement 10**
Should Japan accept foreign workers more actively?
**(Japanese VAA) Statement 11**
Nuclear power plants are necessary for Japan?
**(Japanese VAA) Statement 12**
The Constitution should be revised to specify the Self-Defense Forces.
**(Japanese VAA) Statement 13**
Privacy and individual rights can be restricted to protect public order.
**(Japanese VAA) Statement 14**
Do you agree with lowering the eligibility age for parliamentarians?

For example, statements 4, 6, and 12 pertain to issues that have recently gained considerable importance. The ruling party in Japan has announced its intention to implement new defense and security measures beginning from the latter half of 2021. Experts and people were in an uproar over these initiatives. Such discussions are not novel, whether they concern expanding the defense budget, strengthening military capabilities, or resorting to nuclear weapons. However, in light of the challenging geopolitical contexts in Japan's neighborhood and in the world, these issues have received greater significance and more attention.

Table 6 shows statement-wise suggestions extracted from the YouTube comment data, providing insights into Japanese public opinion on constitutional amendments. Discussion is related to the Self-Defense Forces and Article 9, which is the clause in the Japanese Constitution that renounces war and prohibits Japan from maintaining armed forces with war potential. This clause came into effect in 1947. The debate regarding Article 9 has been ongoing for decades, and there are divided opinions even among political parties. To conduct an amendment, a two-thirds majority vote is required in both houses of the National Diet, followed by a national referendum, highlighting the complexity and difficulty of the issue. The system output the top five suggestions, each reflecting varying perspectives on the issue and accompanied by a similarity score indicating relevance to the original statement. The first suggestion, with the highest similarity score of 0.77, advocates for constitutional amendments to include not only the specification of the Self-Defense Forces but also an Emergency Measures Law and an Espionage Prevention Law, emphasizing the importance of national security. Subsequent suggestions present different views: one supports the inclusion of an emergency clause before addressing Article 9, another shifts focus to broader constitutional changes beyond the Self-Defense Forces, and others express concerns about specific amendments proposed by the LDP, such as the deletion of Article 9. The system-generated suggestions highlight key areas of public discourse and provide valuable context for VAA designers to consider. The obtained results demonstrate the system's ability to generate relevant, statement-wise suggestions from unconventional data sources, such as YouTube comments, which are not typically used by VAA designers. The full version of the system's translated output in PDF format, as well as the implemented code, can be found at https://github.com/DBurya/VAASuggestions_GIQ.

**Table 6:** A translated example of system suggestions for a VAA statement produced based on YouTube comment data.

**VAA statement**
The Constitution should be revised to specify the Self-Defense Forces.
**System suggestion 1**
I believe we need to properly amend the Constitution to include an Emergency Measures Law, an Espionage Prevention Law, and the exercise of the right to self-defense. **Similarity Score: 0.77**
**System suggestion 2**
Personally, I don't think the LDP proposal is that bad, and I think it would be better to have an emergency clause first rather than amend Article 9. I also think it is important to establish a system to properly honor SDF personnel who have died in the line of duty as a nation. **Similarity Score: 0.75**
**System suggestion 3**
The main point is not Article 9 or the Self-Defense Forces, but other parts of the Constitution. **Similarity Score: 0.75**
**System suggestion 4**
I think the Constitution needs to be amended, but I am concerned about the LDP's draft. Specifically, I am worried about the deletion of Article 9, which is the supreme law, and the creation of an emergency clause. If these points can be properly explained to me, I can support the constitutional amendment. **Similarity Score: 0.74**
**System suggestion 5**
I believe that constitutional changes, such as the clarification of the Self-Defense Forces, are necessary. However, I do not think that an emergency clause is necessary. It would be sufficient to interpret and operate the existing provisions as long as they do not violate the public welfare. Keeping it as a request is deliberate and inadequate. **Similarity Score: 0.74**

Discovering information from textual data is a powerful technique recently applied in various disciplines (Jung and Lee, 2020). The current study is the first of its kind in which a machine learning approach was employed to improve the effectiveness of the VAA statement formulation process by extracting valuable suggestions from different data sources. Today's VAAs generally pay little attention to the question of which sources to use when formulating statements. Due to the limited amount of time and resources available, VAA designers' judgments are often based on party manifestos only. Understanding all potentially appropriate VAA policy statements that reflect both the parties' and potential voters' perspectives is beneficial for the statement formulation process; however, it requires substantial manual effort and is still vulnerable to bias. To address these challenges and minimize bias, a system capable of analyzing textual data from various sources and generating relevant statement suggestions was necessary. Although the proposed approach still requires human intervention, it provides opportunities for political analysis, thereby making the statement selection process more transparent and faster. In the case study, YouTube comments and party manifestos were utilized separately to produce statements-wise suggestions. It appears that, in contrast to the stances outlined in party manifestos, people on YouTube pay greater attention to political issues and often express more radical opinions. Results also suggest that topics discussed on YouTube are only partially covered and placed less emphasis on by the political parties. On the other hand, it can be due to the limited number of videos related to politics. The results add more weight to the argument that VAA designers need to use different information sources to formulate statements relevant to both political parties and potential voters. Understanding public sentiment would enhance the effectiveness of VAAs in user engagement. In addition, the system can also be used as a tool to check the relevancy of VAAs.

Table 7 presents that out of all applications, the Asahi Shimbun VAA covers more than 60% of statements produced by the system. This may indicate that this VAA covers issues of concern to both YouTube users and the topics addressed by political parties. Interestingly, the statements covered by FokusJapan's system were exclusively based on party manifestos, unlike other VAAs where, although most statements are predominantly derived from party manifestos, the coverage is not exclusive. This trend, evident across most VAAs as shown in the table, suggests a prevalent reliance on the traditional information sources. While VAAs accurately reflect official party positions, they may not fully capture the informal yet significant discourse among the users on social media platforms like YouTube. The inclusion of comments from other platforms in VAAs could bridge this gap, providing a more holistic view of public opinion. For instance, the Mainichi Shimbun and Shimotsuke Shimbun VAAs incorporate both manifestos and comments, potentially offering a more balanced perspective that encompasses both formal political agendas and public opinions. This approach may enhance the relevance and accuracy of VAAs, making them more reflective of the diverse viewpoints within the electorate.

**Table 7:** The system's verified statements covered by Japanese voting advice applications.

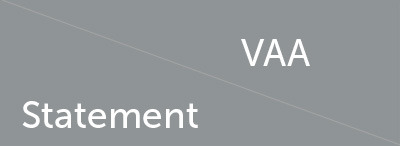	**Mainichi Shimbun**	**Zero Senkyo**	**Asahi Shimbun**	**FokusJapan**	**Japan Choice**	**Shimotsuke Shimbun**
1	Manifestos	×	Comments	×	×	Comments
2	×	×	Comments	Manifestos	Manifestos	×
3	×	×	Comments	×	×	×
4	×	Manifestos	×	×	×	Manifestos
5	Both	Comments	Manifestos	×	Manifestos	×
6	Manifestos	×	Comments	×	Manifestos	Manifestos
7	Comments	×	Comments	Manifestos	×	Manifestos
8	×	Manifestos	×	×	Manifestos	Manifestos
9	×	Manifestos	Manifestos	×	Manifestos	Manifestos
10	×	×	×	Manifestos	Comments	Both
11	Comments	Comments	Manifestos	×	Manifestos	×
12	Manifestos		Manifestos	×	Both	Comments
13	Manifestos		Manifestos	Manifestos	Manifestos	×
14	Manifestos		×	Manifestos	×	Manifestos
15	Manifestos		×	Manifestos	×	Manifestos
16	×		×	×	×	Both
17	Manifestos		Manifestos	×		×
18	Manifestos		×	Manifestos		×
19	Manifestos		Comments	×		
20	×		Manifestos	×		
21	×		×			
22	Manifestos		Manifestos			
23	×		Manifestos			
24	Comments		Manifestos			
25	Both		×			

Table 8 demonstrates short versions of statements not selected by the system. Initially, 52 statements were identified as missing, not achieving a cosine similarity threshold of 0.5. These were then narrowed down to 14 after recognizing overlaps in topics, allowing for a more precise representation of the main areas overlooked by the system. It helped to find key thematic gaps in the system's coverage, such as digital administration, socioeconomic policies, and electoral processes. The absence of these topics in the system's output shows potential areas for improvement in ensuring a comprehensive scope that would address all significant public concerns. To fill these gaps, utilizing more diverse datasets is crucial, as these statements were not adequately discussed in either party manifestos or YouTube comments. This highlights the need for ongoing updates to include a broader range of topics relevant to current public discourse and policy debates.

**Table 8:** Representative statements not produced by the system.

Should public document management be rigorously enforced?	Should resident foreigners be given voting rights?
Do you support enabling online voting in elections?	Should imperial succession be limited to male-line males only?
Do you support the government's goal to increase men's paternity leave uptake to 30% by 2025?	Should domestic industries be protected while promoting trade and investment liberalization?
Should the merging of electoral districts in the House of Councilors be allowed to correct vote disparities?	Do you support selling emergency contraception without a prescription in pharmacies?
Should companies redistribute their retained earnings more broadly?	Should the GoTo campaign be restarted early?
Should the minimum wage be raised to at least 1,500 yen?	Should a basic income be guaranteed?
What are your thoughts on managing administration using digital technology and My Number Card?	Should the Moritomo/Kake Gakuen and Cherry Blossom Viewing issues be re-investigated?

## 7 Conclusions

In this work, a framework and system was proposed to assist the design of VAAs and alleviate the load of the VAA designers by generating statement-wise suggestions for VAA designers from different data sources. The research demonstrated the system's ability to employ methods based on the documents' characteristics and VAA designers' requirements to facilitate the statement formulation process. The research contributes to the domain by providing a reproducible support solution for designing voting advice applications on one of the expensive elements, which has been traditionally performed by humans.

However, although the system's results were tested in a real-world scenario and demonstrated substantial improvements, it has multiple limitations. The methods utilized in the system are built on the state-of-the-art BERT language model. It is to note, however, that BERT also has its drawbacks. For example, it has redundant parameters. Kovaleva et al. (2019) found that BERT is highly over-parameterized, which led to the development of optimized BERT models such as ELECTRA, RoBERTA, DistilBERT, etc. Despite this, the original BERT was incorporated into the proposed system since it consistently produces reliable results in natural language processing tasks (Acheampong et al., 2021).

BERT topic modeling is another area of improvement. It produced coherent topics with documents; some documents were, however, identified as outliers due to their inability to be assigned to any topic. Despite this, the outlier documents may still contain valuable information for VAA designers. Further testing in collaboration with VAA designers is necessary to ensure the practicality and usefulness of the system.

While the primary objective of the proposed system was to reduce the cost of designing VAA statements, it has several other possible uses. Policy statements determine the quality of a VAA. Hence it is important to test them before or in the early stages of their publication online. The system would allow statement verification by exploring their relevance to the upcoming elections. The ability of the system to analyze relevancy using a two-sided approach has an advantage compared to the previous approaches: the ability to verify a large amount of potentially appropriate VAA policy statements that reflect both the parties' and the potential voters' political views.

Most of the VAAs around the world are still designed by humans that makes them dependent on the human factor. An information technology-based tool was therefore proposed to at least partially alleviate this problem. Nevertheless, an open question is to what extent one should utilize the various datasets. Employing the datasets—such as party manifesto, social media and e-petition (Buryakov et al., 2022b) data separately, would create a similar problem of an excessive amount of textual data that VAA designers would have to go through. In future work, research can focus on addressing these challenges and also on extending the system to automatically generate utterly new VAA statements, thus further facilitating VAA designers' work. This advancement would help make the VAA statement formulation process more efficient and potentially less biased.

## Data availability statement

The data used in the study are included in the link: YouTube Comments and Party Manifestos (Japanese), and VAA Statements (English and Japanese) - Mendeley Data. Further inquiries can be directed to the corresponding author.

## Author contributions

DB: Conceptualization, Data curation, Methodology, Software, Writing – original draft, Writing – review & editing. MK: Conceptualization, Methodology, Supervision, Writing – review & editing. US: Conceptualization, Methodology, Supervision, Writing – review & editing. VK: Supervision, Writing – review & editing.
